# Supplementary material for: Three-dimensional self-assembly using dipolar interaction
Source: Sci Adv. 2020 May 8;6(19):eaba2007. doi: 10.1126/sciadv.aba2007 (PMC7209989; doi:10.1126/sciadv.aba2007)
Supplement: aba2007_SM.pdf [file aba2007_SM.pdf]

Science Advances
AAAS

advances.sciencemag.org/cgi/content/full/6/19/eaba2007/DC1

Supplementary Materials for

Three-dimensional self-assembly using dipolar interaction

Leon Abelmann*, Tijmen A. G. Hageman, Per A. Löthman, Massimo Mastrangeli, Miko C. Elwenspoek

*Corresponding author. Email: l.abelmann@utwente.nl

Published 8 May 2020, *Sci. Adv.* **6**, eaba2007 (2020)
DOI: 10.1126/sciadv.aba2007

The PDF file includes:

Supplementary Materials and Methods
Figures and Movies
Table S1
Figs. S1 to S3
Legends for movies S1 and S2

Other Supplementary Material for this manuscript includes the following:

(available at advances.sciencemag.org/cgi/content/full/6/19/eaba2007/DC1)

Movies S1 and S2
Zip file with object designs in STL format

Supplementary Material

I. MATERIALS AND METHODS

Table S1 lists the dimensions of the objects used in this experiment. The STL files used for 3D printing are attached in the file `Designs.zip`.

TABLE S1: *Outer dimensions of the objects investigated, the value between brackets is the standard deviation in units of the last digit, estimated from four measurements.*

	ΔE (μJ)	Aspect Ratio	Diameter (mm)	Height (mm)
Spheroid	-40	1.0	18.3(1)	19.1(1)
	0	1.3	14.6(1)	19.1(1)
	+40	1.6	14.6(1)	23.6(2)
Cylinder	-40	1.0	18.7(1)	19.2(1)
	0	1.3	14.8(1)	19.2(1)
	+40	1.6	14.8(1)	23.8(1)
Cube	-40	1.0	18.5(1)	19.2(1)
	0	1.3	14.6(1)	19.2(1)
	+40	1.6	14.6(1)	23.7(1)

II. FIGURES AND MOVIES

Figure S3 shows the effect of increasing amount of objects. Videos of the effect of shape (Figure S1) and the effect of the number of objects (Figure S2) are available as additional material and online (underlined links).

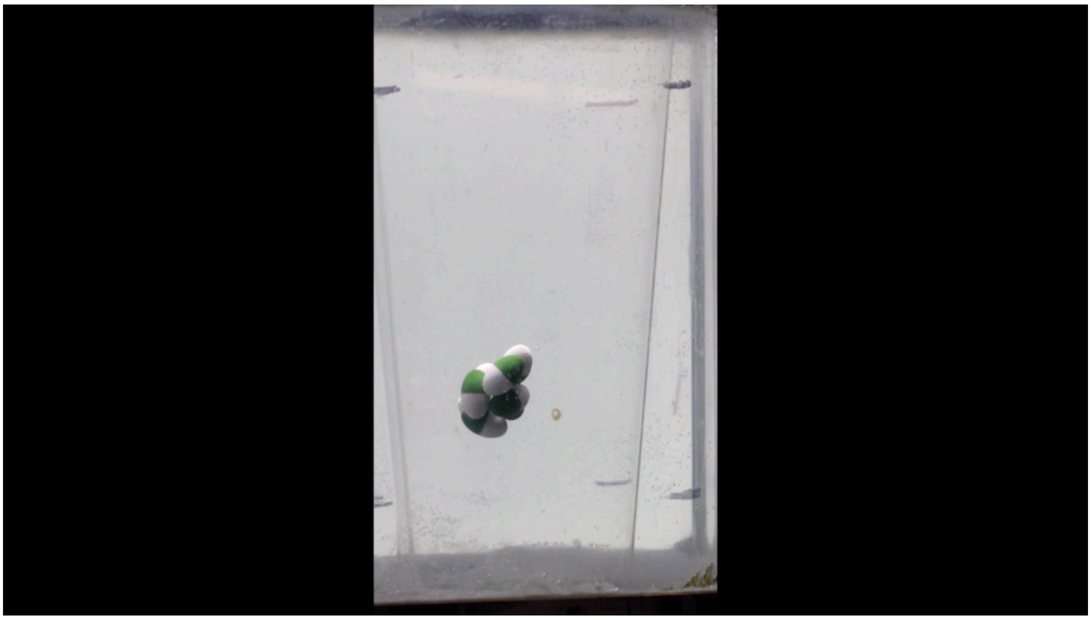

FIG. S1: `ClusterGrowthShape.mp4`*: Video recording of eight spheroids, cylinders, and spheroids with three different aspect ratios*

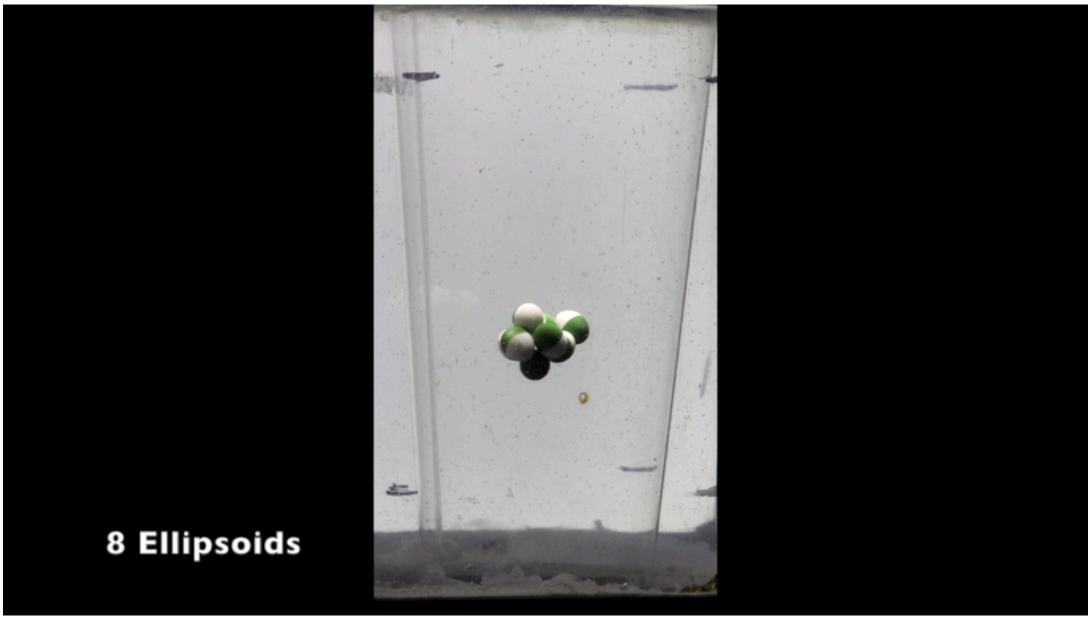

FIG. S2: `ClusterGrowthNumberParticles.mp4`*: Video recording Video recording of spheroids, cylinders, and spheroids with balanced energy for the parallel and anti-parallel alignment, with 8, 12, and 16 objects.*

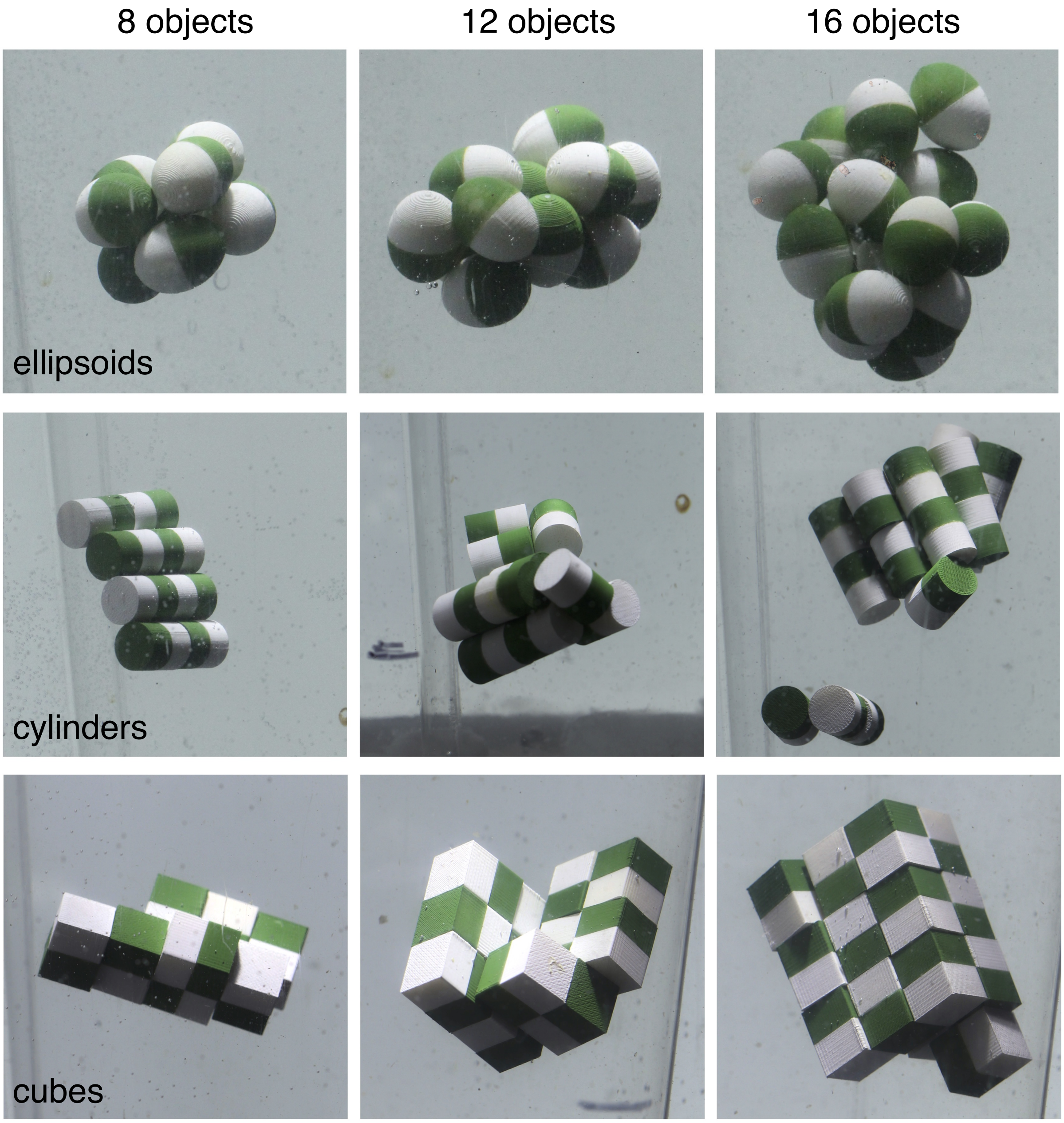

FIG. S3: . *Increasing the number of objects. Using an object shape for which the parallel and anti-parallel configuration are identical, the number of objects in the cylinder is increased from 8 to 16 (left to right). The spheroids in the top row do not form regular structure. The cylinders and cubes tend to form crystals, with an occasional "adatom" on the outside that follows a flux-closure structure.*

Movie S1: Video recording of eight spheroids, cylinders, and spheroids with three different aspect ratios.

Movie S2: Video recording of spheroids, cylinders, and spheroids with balanced energy for the parallel and anti-parallel alignment, with 8, 12, and 16 objects.
